# Ginsenoside Rg1 Ameliorates Palmitic Acid-Induced Hepatic Steatosis and Inflammation in HepG2 Cells via the AMPK/NF-*κ*B Pathway

**DOI:** 10.1155/2019/7514802

**Published:** 2019-07-28

**Authors:** Qing Xiao, Shujun Zhang, Cheng Yang, Ruoyang Du, Jinqiu Zhao, Jiajun Li, Yashu Xu, Yuanyuan Qin, Yue Gao, Wenxiang Huang

**Affiliations:** ^1^Chongqing Key Laboratory of Infectious Diseases and Parasitic Diseases, Department of Infectious Diseases, The First Affiliated Hospital of Chongqing Medical University, Chongqing, China; ^2^Department of General Medicine, People's Hospital of Chongqing Bishan District, Chongqing, China; ^3^Department of Infectious Diseases, Chongqing Public Health Medical Center, Chongqing, China

## Abstract

Nonalcoholic fatty liver disease (NAFLD) is one of the common diseases in the world, and it can progress from simple lipid accumulation to sustained inflammation. The present study was designed to investigate the effects and underlying mechanisms of ginsenoside Rg1 (G-Rg1) treatment on NAFLD* in vitro*. HepG2 cells were treated with palmitic acid (PA) to induce steatosis and inflammation and then successively incubated with G-Rg1. Lipids accumulation was analyzed by Oil Red O staining and intracellular triglyceride (TG) quantification. Inflammatory conditions were examined by quantifying the levels of cell supernatant alanine transaminase/aspartate aminotransferase (ALT/AST) and secretory proinflammatory cytokines, including IL-1*β*, IL-6, and TNF-*α* in the cell supernatants. Quantitative RT-PCR and western blotting were used to measure the expressions of genes and proteins associated with lipogenic synthesis and inflammation, including AMP-activated protein kinase (AMPK) and nuclear factor-kappa B (NF-*κ*B) pathways. HepG2 cells were pretreated with an AMPK inhibitor; then, Oil Red O staining and TG quantification were performed to study the lipid deposition. Phospho-AMPK (Thr172) (p-AMPK) and phospho-acetyl-CoA carboxylase (Ser79) (p-ACC*α*) were quantified by immunoblotting. Immunofluorescence was performed to demonstrate the nuclear translocation of NF-*κ*B P65. The present study showed that PA markedly increased the intracellular lipid droplets accumulation and TG levels, but decreased AMPK phosphorylation and the expressions of its downstream lipogenic genes. However, G-Rg1 alleviated hepatic steatosis and reduced the intracellular TG content; these changes were accompanied by the activation of the AMPK pathway. In addition, blocking AMPK by using the AMPK inhibitor markedly abolished the G-Rg1-mediated protection against PA-induced lipid deposition in HepG2 cells. Furthermore, G-Rg1 reduced the ALT/AST levels and proinflammatory cytokines release, which were all enhanced by PA. These effects were correlated with the inactivation of the NF-*κ*B pathway and translocation of P65 from the cytoplasm to the nucleus. Overall, these results suggest that G-Rg1 effectively ameliorates hepatic steatosis and inflammation, which might be associated with the AMPK/NF-*κ*B pathway.

## 1. Introduction

Nonalcoholic fatty liver disease (NAFLD) is a serious public health issue in well-off countries with a prevalence of 25%-46.2%; it is related to hyperlipidemia, type-2 diabetes, cardiovascular diseases, and metabolic syndrome [1, 2]. This liver disease follows a series of steps, extending from hepatic steatosis (simply the accumulation of triglycerides (TGs) in the liver), steatohepatitis (steatosis with inflammation), and fibrosis to cirrhosis and, ultimately, hepatocellular carcinoma [3]. Although the mechanisms underlying the progression of steatosis towards more aggressive stage of NAFLD still remain largely unknown, the ‘two-hit' hypothesis has been widely accepted. Aggregation of lipids in the cytoplasm of liver cells (first hit) brings about a series of cytotoxic events (second hit), resulting in liver inflammation [4]. Currently, no approved medication for NAFLD treatment is available, other than lifestyle advice on diet and exercise. Thus, there is an extremely urgent need to find new therapies [5].

AMP-activated protein kinase (AMPK) is a heterotrimeric serine/threonine kinase that is made up of a catalytic *α* subunit together with two regulatory subunits, *β* and *γ* [6]. AMPK complexes are taken as an energy sensor regulating glucose and lipid metabolism in various organs including the liver and adipose tissue; the activities of these complexes are mediated by changes in the cellular AMP:ATP or ADP:ATP ratios and by some commonly used diabetes medications, such as metformin and thiazolidinediones [7]. Moreover, the biochemical activities of AMPK are firmly regulated by phosphorylation or dephosphorylation by upstream kinases and phosphatases, respectively [8]. AMPK phosphorylation causes the inhibitory expression of downstream key genes in fatty acid* de novo* synthesis, which are targets of AMPK in the liver or adipose tissue, including sterol regulatory element binding proteins (SREBPs), fatty acid synthase (FAS), and acetyl-CoA carboxylase (ACC). Upon activation, AMPK enhances fatty acid oxidation and decreases the productions of glucose, TGs, and cholesterol. Consequently, AMPK improves lipid and glucose homeostasis, making it a tempting therapeutic target for treating metabolic syndromes, including type 2 diabetes [9, 10]. Efforts are continuously being taken by many pharmaceutical companies to discover direct AMPK activators for the cure of metabolic diseases, such as NAFLD and cardiovascular diseases [11].

In hepatocytes, transcriptional regulation of inflammation-related genes is controlled by well-documented transcription factors. Nuclear factor-kappa B (NF-*κ*B) is considered to play a crucial role in the transcription of proinflammatory cytokines [12]. This nuclear factor is usually activated after phosphorylation and the subsequent degradation of its inhibitor I*κ*B [13]. Studies have found that when cells are stimulated by LPS, carbon tetrachloride, or palmitic acid (PA), the p65 subunit of NF-*κ*B is activated in the cytoplasm; it then enters the nucleus and specifically binds to its target DNA sequence, thereby regulating the expression of its target proinflammatory genes, including tumor necrosis factor-*α* (TNF-*α*), interleukin-1 beta (IL-1*β*), and interleukin-6 (IL-6). Thus, NF-*κ*B plays a key role in important physiological and pathological processes such as immunity, aging, inflammation and tumor formation [14, 15]. Liver-specific activation of NF-*κ*B causes the elevated synthesis of proinflammatory mediators. Simultaneously, the pharmaceutical inhibition of NF-*κ*B activation is associated with improvement of metabolic dysfunction in the liver. Moreover, NF-*κ*B itself and its target genes also represent important targets for drug screening and disease treatment [16].

**Figure 1:**
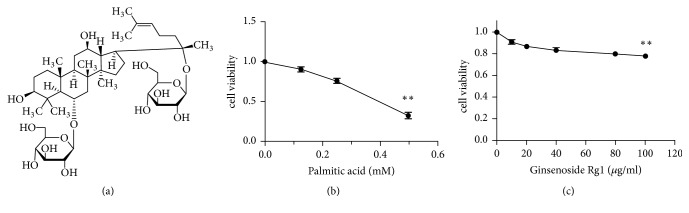
*Viability of HepG2 cells after exposure to palmitic acid (PA) or ginsenoside Rg1 (G-Rg1).* The structure of G-Rg1. (a) HepG2 cells were exposed to various concentrations of PA (0, 0.125, 0.25, and 0.5 mM) and G-Rg1 (0, 10, 20, 40, 80, and 100 *μ*g/mL). After being incubated with these compounds for 24 h, the cell viability was determined by the CCK-8 assay. (b, c) The data represent the means ± SDs of three independent experiments. *∗∗P*<0.01 (in comparison to the vehicle-treated control group).

Panax ginseng is one of the oldest traditional Chinese medicinal herbs; it has been reported to have anti-ischemic myocardial injury [17], anti-Alzheimer's disease [18], antidiabetic effects [19] and other similar activities [20]. The active ingredients of ginseng are thought to be ginsenosides. Presently, more than 40 types of ginsenosides have been identified. Among these ginsenosides, Rg1 (Figure 1(a)) is one of the most active, even though it is found only in trace amounts [21]. In recent years, it has been reported to play a protective role in various liver diseases, including alcoholic hepatitis, acute liver failure, and liver fibrosis [22–24]. Our previous study has shown that Rg1 can alleviate NAFLD by improving lipid peroxidation, reducing endoplasmic reticulum stress, and inhibiting inflammasome activation in high-fat induced NAFLD mouse [25]. However, the role of Rg1 in lipid deposition, which is the initiation process of NAFLD, is unknown; the possible direct targets in the subsequent more severe inflammatory responses are not well understood either. Therefore, in this study, we examined the PA-induced lipid deposition in HepG2 cells following Rg1 treatment to evaluate the anti-lipid deposition and anti-inflammatory effects of Rg1* in vitro* and explore its possible molecular mechanisms.

## 2. Materials and Methods

### 2.1. Cell Culture

The HepG2 human hepatoma cell line was purchased from Procell (Wuhan, China). HepG2 cells were cultured in Dulbecco's modified Eagle's medium (DMEM) (Hyclone, Logan, UT, USA) containing 10% fetal bovine serum (FBS) (GIBCO, Grand Island, New York) and 1% penicillin/streptomycin (Beyotime, Shanghai, China) in a humidified atmosphere containing 5% CO2 at 37°C. The cells were seeded in six-well plates at the density of 1.2×10^6^ cells/well and were allowed to adhere overnight; they were then cultured with fatty-acid-free bovine serum albumin (BSA) (Solarbio, Beijing, China) or palmitic acid (PA) (Sigma-Aldrich, St. Louis, MO, USA) in culture medium for 24 h. The cells were washed thrice with sterile phosphate buffered saline (PBS) (Hyclone) and were either left untreated, or pretreated with the AMPK inhibitor, Dorsomorphin (Compound C) (Selleck, USA) for 1 h prior to treatment with ginsenoside Rg1 (G-Rg1) (Meilunbio, Dalian, China) dissolved in 0.1% dimethyl sulfoxide (DMSO) (Sigma-Aldrich) for 6 h.

### 2.2. Cytotoxicity Assay

HepG2 cells were seeded in 96-well plates at the density of 1.5 × 10^4^ cells/well and cultured in 100 *μ*L of DMEM containing 10% FBS overnight. The medium was then discarded and was replaced with 0, 0.125, 0.25, and 0.5 mM PA or 0, 10, 20, 40, 80, and 100 *μ*g/mL G-Rg1 diluted in the culture medium, followed by incubation for a further 24 h. Subsequently, cell viability was assessed using Cell Counting Kit-8 Assay (CCK-8; Dojindo, Tokyo, JAPAN), according to the manufacturer's instructions. The cells were incubated with 10% CCK-8 solution for another 2 h at 37°C in the dark, and the absorbance of the sample in each well was measured at 450 nm using a Varioskan Flash Microplate Reader (ThermoFisher Scientific, Waltham, MA, USA). Each experiment was independently repeated thrice.

### 2.3. Intracellular TG Measurement

Intracellular TG levels were assayed using a Triglyceride Assay Kit (Solarbio) following the manufacturer's instructions.

### 2.4. Oil Red O Staining

HepG2 cells were seeded in six-well plates (1.2×10^6^ cells/well), treated with 0.25 mM PA, and then cultured with the previously indicated concentrations of G-Rg1 for another 6h. The cells were washed thrice with PBS and fixed with 4% neutral formaldehyde in PBS for 30min. Then they were washed thrice with double distilled water and stained with freshly prepared Oil Red O solution (1*μ*g/mL, Oil Red: deionized water = 3:2) for 20 min at room temperature. After staining, the cells were thoroughly washed with double distilled water to remove the unbound staining solution and visualized using a microscope (Olympus, Tokyo, Japan); the cells were then photographed. To quantify the Oil Red O contents, isopropanol was added to each well and the optical density of the samples at a wavelength of 450 nm was measured using a microplate reader.

### 2.5. Biochemistry Analysis

The cell supernatant levels of alanine transaminase (ALT) and aspartate aminotransferase (AST) in the supernatant were monitored using standard clinical chemistry assays on Automated Chemistry Analyzer (Mindray, Shenzhen, China).

### 2.6. Enzyme-Linked Immunosorbent Assay (ELISA)

Assays for proinflammatory markers were carried out by means of commercial enzyme-linked immunosorbent assay (ELISA) kits according to the manufacturer's instructions; ELISA kits for the following molecules were used: human IL-1*β*, IL-6, and TNF-*α* (all from Neobioscience, Shenzhen, China). First, the cell supernatants were added to microplate wells, which were precoated with specific antibodies in advance. After 1.5 h of incubation at 37°C, the unbound supernatants were washed away, and enzyme-linked antibodies were added to each well, followed by incubation for 1 h at 37°C. Then, a second washing step was performed to remove the unbound reagents. Finally, the enzyme-binding diluent was pipetted into each well, followed by incubation for 30 min, and the stop solution was added to end the color development reaction. The absorbance of the samples at 450 nm was measured using the microplate reader. A standard curve based on the results obtained using eight human standard solutions was plotted, and the sample concentrations were confirmed. All assays were performed in triplicate for each experiment.

### 2.7. Quantification Real-Time PCR

Total RNA was isolated using the High Purity Total RNA Rapid Extraction Kit (BioTeke, Beijing, China) according to the manufacturer's instructions, and the absorbances of the extracted RNAs at 260 nm and 280 nm were determined spectrophotometrically (Molecular Devices, Ramsey MN USA). RNA samples with purity ratios (A260/A280) between 1.8 and 2.0 were used for synthesizing single-strand cDNA by means of All-in-One cDNA Synthesis SuperMix (Bimake) for quantitative real-time polymerase chain reaction (qPCR). Gene-specific primers (Table 1) were designed by Invitrogen (Carlsbad, CA, USA). qPCR was performed to detect the relative mRNA expression levels using 2 ×SYBR Green qPCR Master Mix (Bimake) as described in the protocol. To determine the specificity of the amplification, melting curve analysis was performed for all final PCR products. Relative changes in gene expression were calculated and expressed as fold changes using the relative quantification (ΔΔCt) method. The relative abundance of each transcript was normalized to that of *β*-actin. Each sample was run in triplicate.

### 2.8. Western Blotting

HepG2 cells were harvested and resuspended in RIPA lysis buffer (Wanleibio) containing 1% protease inhibitor (Beyotime) and 1% phosphatase inhibitors (Beyotime) for 10 min at 4°C, after which they were sonicated thrice (15 seconds each) with the Ultrasonic crusher (SANYO, UK). The cell lysates were centrifuged at 14,000×g for 15 min at 4°C, and then the supernatants were collected; the protein concentrations in the supernatants were quantified using the BCA Protein Quantification Kit (KeyGEN BioTECH, Jiangsu, China). Equal amounts of the lysate proteins (20*μ*g/lane) were separated by 10% sodium dodecyl sulfate–polyacrylamide gel electrophoresis (SDS-PAGE) (Bio-Rad, Hercules, CA, USA); the protein bands were then transferred onto polyvinylidene fluoride (PVDF) membranes (Merck Millipore, UK). The membranes were blocked by using 5% nonfat milk powder in PBS (BOSTER, Wuhan, China) containing 0.1% Tween-20 (Bio-Rad) (PBS-T), and then the membranes were incubated with the specific primary antibodies overnight at 4°C. Thereafter, the membranes were washed thrice with PBS-T and incubated with HRP-conjugated secondary rabbit antibodies (1:5000, Abbkine, CA, USA) at room temperature for 1 h. In this experiment, the endogenous loading control used was GAPDH. The primary antibodies used included anti-AMPK, anti-p-AMPK (Thr172), anti-ACC*α*, anti-p-ACC*α* (Ser79), anti-FAS, anti-I*κ*B*α*, anti-p-I*κ*B*α* (Ser32), anti-NF-*κ*B, anti-p-NF-*κ*B (Ser526), anti-IL-1*β*, anti-IL-6, and anti-TNF-*α* antibodies (1:1000, all from Cell Signaling Technology, Beverly, MA, USA) and anti-SREBP-1c and anti-GAPDH antibodies (both from Wanleibio). The immunoblots were visualized using an enhanced chemiluminescence solution (GE Healthcare, Buckinghamshire, United Kingdom), and the bands were visualized using a chemiluminescence imaging system (Bio-Rad). The intensity of the individual band was quantified using the Image Lab 6.0 software. The western blotting analysis was performed in triplicate.

### 2.9. Immunofluorescence

The cells subjected to various treatments (in 24-well plates) were rinsed thrice with PBS and fixed using 4% cold PBS-buffered paraformaldehyde, followed by permeabilization with 0.2% Triton X-100. The cells were then incubated and blocked with the blocking solution (5% BSA/PBS), followed by incubation overnight at 4°C with the primary antibody—rabbit anti-NF-*κ*B (1:1000, Cell Signaling Technology). Thereafter, the cells were incubated with Alexa Fluor® 488-Conjugated Goat anti-Rabbit IgG (H+L) (1:100,ZSGB-BIO, China) for 1 h in the cassette after washing them with PBS; they were then dyed with 10 *μ*g/mL ready-to-use DAPI solution (Solarbio) for 3 min to stain the nuclei. Representative areas were photographed using a laser scanning confocal microscope (Zeiss, Germany). The experiment was repeated thrice.

### 2.10. Statistical Analysis

All results are expressed as the means ± standard deviations (SDs). Statistical analysis was performed using one-way ANOVA where appropriate, followed by performance of Tukey's post hoc test. Statistical significance was considered for* P* values < 0.05.

## 3. Results

### 3.1. Viability of HepG2 Cells after Exposure to PA or G-Rg1

Before conducting the main experiment, we evaluated the effect of PA and G-Rg1 on HepG2 cell viability using the CCK-8 assay. We discovered that PA did not show significant cytotoxicity at concentrations no more than 0.25 mM, and the cell viability decreased sharply by 67.4% following treatment with 0.5 mM PA (Figure 1(b)). Likewise, G-Rg1 was nontoxic until up to a concentration of 100 *μ*g/mL, at which the cell viability was reduced by 22% (Figure 1(c)). Thus, 0.25 mM PA and 40 and 80 *μ*g/mL G-Rg1, which were found to be nontoxic to HepG2 cells, were used for subsequent experiments.

### 3.2. G-Rg1 Inhibits Lipid Accumulation in HepG2 Cells

**Figure 2:**
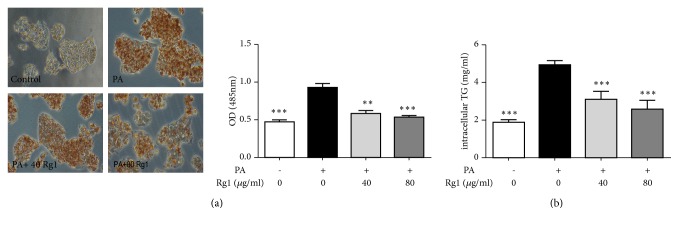
*Inhibitory effect of G-Rg1 on PA-induced lipid accumulation in HepG2 cells. *HepG2 cells were treated with 0.25 mM PA for 24 h and then cultivated with 40 or 80 *μ*g/mL G-Rg1 for 6 h. The lipid accumulation was shown by Oil Red O staining (original magnification × 200) and the absorbance of the samples at 450 nm was measured using a microplate reader. (a) Effect of G-Rg1 on the intracellular triglyceride (TG) content in PA-treated HepG2 cells. (b) The data are expressed as the means ± SDs; n=3; *∗P*<0.05, *∗∗∗P*<0.001 (in comparison to the PA-treated model group).

The* in vitro *model of PA-induced hepatic steatosis model was used to investigate the efficacy of G-Rg1 against hepatic steatosis. Oil Red O staining indicated that PA treatment for 24 h significantly induced significant intracellular lipid accumulation in HepG2 cells. The absorbance of the staining cells in the PA-treated model group was 1.94 times that of the cells in the control group. However, the intracellular lipid accumulation was reduced by G-Rg1 in a concentration-dependent manner; 40 *μ*g/mL and 80 *μ*g/mL of G-Rg1 reduced the absorbances of the model group samples by 36.8% and 42.0%, respectively (Figure 2(a)). Simultaneously, the G-Rg1-mediated inhibition of lipid accumulation in HepG2 cells was assessed by the quantification of intracellular TGs. The intracellular TG accumulation promoted by PA rose by 1.62 times; this was later lowered by 36.7% following treatment with 40 *μ*g/mL G-Rg1 and by 47.9% following treatment with 80 *μ*g/mL G-Rg1(Figure 2(b)).

### 3.3. G-Rg1 Reduces the Levels of Biochemical Indicators in HepG2 Cells

As shown in Table 2, the levels of ALT and AST in the cell supernatant were significantly higher in the model group than those in the control group. However, treatment with 40 *μ*g/mL and 80 *μ*g/mL G-Rg1 decreased the ALT and AST levels in a dose-dependent manner.

### 3.4. G-Rg1 Reduces the Release of Proinflammatory Cytokines in HepG2 Cells

**Figure 3:**
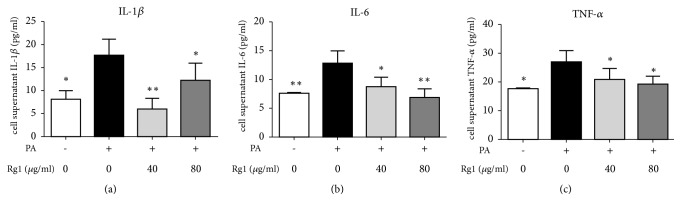
*The effect of G-Rg1 on the release of proinflammatory cytokines in PA-treated HepG2 cells*. HepG2 cells were treated with 0.25 mM PA for 24 h and then cultivated with 40 or 80 *μ*g/mL G-Rg1 for 6 h. The levels of IL-1*β* (a), IL-6 (b), and TNF-*α* (c) in the cell supernatants were measured. The data are presented as the means ± SDs; n=3; *∗P*<0.05, *∗∗P*<0.01 (in comparison to the PA-treated model group).

Previous studies have indicated that NAFLD induces inflammation and promotes the release of some cytokines, such as IL-1*β*, IL-6, and TNF-*α* [26]. In the current study, the levels of IL-1*β*, IL-6, and TNF-*α* in the cell supernatants increased markedly, i.e., by 1.18, 0.69, and 0.53 times, respectively, in the PA-treated group, as compared to the control group. After G-Rg1 treatment, the levels of proinflammatory cytokines decreased notably. Treatment with a low-dose of G-Rg1 decreased the release of IL-1*β*, IL-6, and TNF-*α* by 66.3%, 31.5%, and 22.6%, respectively; treatment with a high-dose of G-Rg1 decreased the release of IL-1*β*, IL-6, and TNF-*α* by 30.5%,45.9%, and 29.2% (Figure 3).

### 3.5. G-Rg1 Attenuates Lipogenic mRNA and Protein Expressions Induced by PA

**Figure 4:**
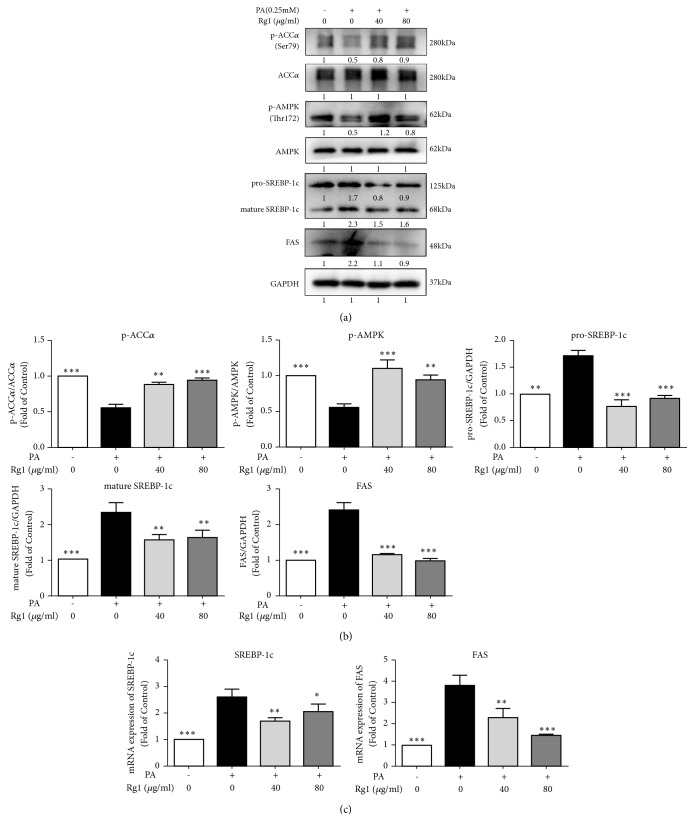
*G-Rg1 reduces the PA-induced increases in the mRNA and protein expression levels of lipogenic genes.* HepG2 cells were treated with 0.25 mM PA for 24 h and then cultivated with 40 or 80 *μ*g/mL G-Rg1 for 6 h. Total proteins were extracted for western blotting (a), quantified by means of the intensities of their bands (b), and total RNA was isolated for qPCR analysis of SREBP-1c and FAS (c). The data represent the means ± SDs of three individual experiments; n=3; *∗P*<0.05, *∗∗P*<0.01, *∗∗∗P*<0.001 (in comparison to the PA-treated model group).

A number of previous studies have reported that AMPK plays an indispensable role in NAFLD, and that its downstream genes—ACC*α*, SREBP-1c, and FAS—are essential for* de novo *fatty acid synthesis [3, 8, 9]. To investigate how G-Rg1 ameliorated fat deposition in the liver, we examined AMPK activity and the expressions of genes associated with lipid metabolism in HepG2 cells. As shown in Figure 4, PA decreased the phosphorylation of AMPK and ACC*α* by 45.3% and 45.7%, respectively, while different concentrations of G-Rg1 markedly enhanced their phosphorylation by 70.9% and 61.9%, respectively. Pro-SREBP-1c, activated-SREBP-1c, and FAS were upregulated by 73.4%, 1.3 times, and 1.4 times following treatment with 0.25mM PA, while G-Rg1 counteracted these PA-induced increases by at least 46.5%, 29.6%, and 52.8%, respectively. The mRNA expressions levels of lipogenic genes, such as SREBP-1c and FAS, increased by 1.6 times and 2.8 times following PA treatment. In contrast, 40 *μ*g/mL G-Rg1 significantly decreased the mRNA levels of SREBP-1c and FAS by 34.5% and 39.2%, compared with the model group. Similarly, 80 *μ*g/mL G-Rg1 reduced the gene expressions of SREBP-1c and FAS by 21.1% and 61.5%, respectively (Figure 4(c)).

### 3.6. The G-Rg1-Mediated Reduction of Lipid Deposition Is Associated with AMPK Activation

**Figure 5:**
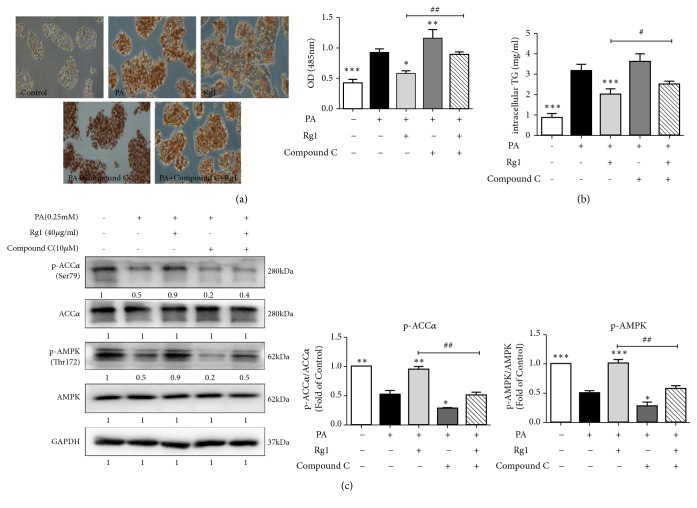
*The G-Rg1-mediated reduction of lipid accumulation is associated with AMPK activation*. HepG2 cells were treated with 0.25 mM PA for 24 h, then cultured with 10 *μ*M Compound C for 1 h, and then cultivated with 40 or 80 *μ*g/mL G-Rg1 for 6 h. The lipid accumulation was examined by Oil Red O staining (original magnification × 200), and the absorbance of the samples at 450 nm was measured using a microplate reader. (a) Effect of G-Rg1 on the intracellular TG levels in PA-treated HepG2 cells. (b) The phosphorylation of AMPK and ACC*α* was examined and quantified by western blotting. (c) The data are expressed as the means ± SDs; n=3; *∗P*<0.05, *∗∗P*<0.01, *∗∗∗P*<0.001 (in comparison to the PA-treated model group).

To identify whether G-Rg1 acts via AMPK, HepG2 cells were pretreated with the selective AMPK inhibitor Compound C (10 *μ*M) for 1 h. Then, the cells were incubated with 0.25 mM PA for an additional 24 h and were either left untreated, or treated with 40 *μ*g/mL G-Rg1 for another 6 h. Lipid accumulation was examined by Oil Red O staining, which dyes lipid droplets red in HepG2 cells. The resultant absorbance, indicative of the intracellular lipid levels, revealed that G-Rg1 significantly attenuated PA-induced lipid accumulation, with the cell absorbance decreasing by 36.8%. Moreover, Compound C significantly reversed the effect of G-Rg1-mediated reduction of lipid deposition, with the cell absorbance increasing by 50.8% (Figures 5(a) and 5(b)). Similarly, the intracellular TG levels were lowered by 36.2% after G-Rg1 treatment, compared with the PA-cultivated group, which was enhanced by 2.9 times following PA treatment (Figure 5(c)). Consistent with previous results, the phosphorylation of AMPK and ACC*α* was reduced in PA-treated HepG2 cells, compared with the cells in the control group. Moreover, Compound C abolished the effects of G-Rg1 on AMPK and ACC*α* phosphorylation. The phosphorylation of both AMPK and ACC*α* in cells treated with PA and Compound C was decreased by 60.0%, compared to that in cells subjected to PA treatment alone. As predicted, pretreatment with Compound C decreased the phosphorylation of AMPK and ACC*α* by 44.4% and 55.6%, respectively. These results are consistent with those demonstrated in Figure 4 and indicate that G-Rg1 antagonized PA-induced hepatic lipid accumulation via the activation of AMPK signaling pathway.

### 3.7. The Anti-Inflammatory Effect of G-Rg1 Is Related to NF-*κ*B Inactivation

**Figure 6:**
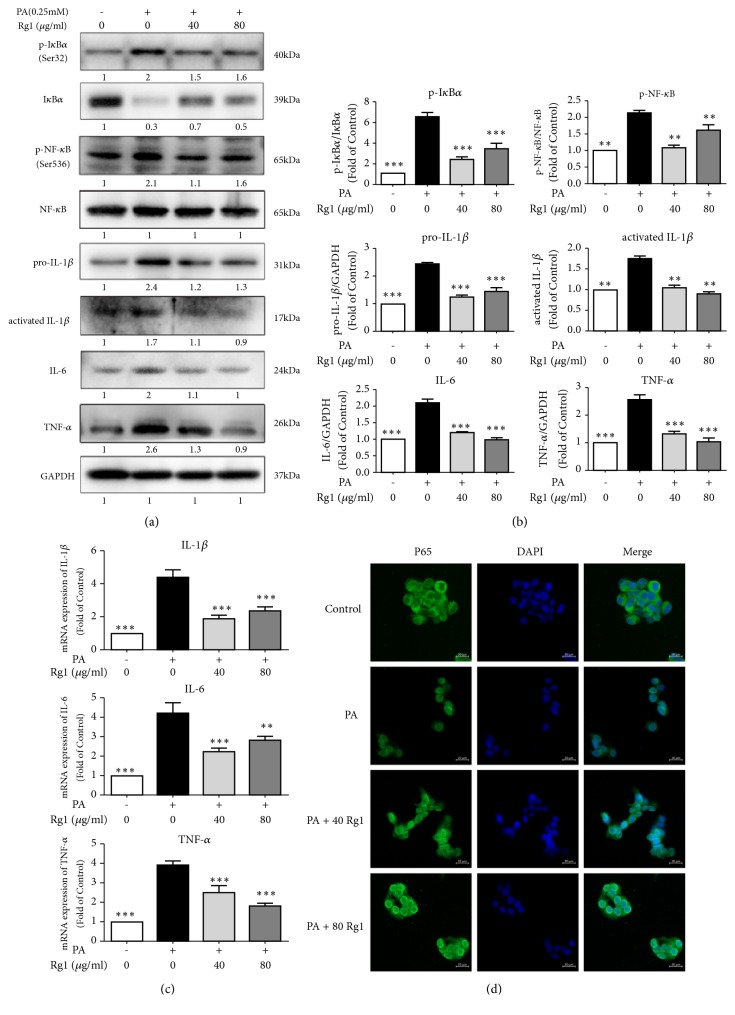
*G-Rg1 attenuates PA-induced liver inflammation through the NF-κB pathway. *HepG2 cells were treated with 0.25 mM PA for 24h and then cultivated with 40 or 80 *μ*g/mL G-Rg1 for 6 h. The cell extracts were subjected to western blotting analysis. (a) The bands intensities were quantified using the Image Lab 6.0 software. (b) The expression levels of inflammatory genes were analyzed using qPCR analysis. (c) The nuclear translocation of NF-*κ*B P65 was observed using laser confocal microscope (scale bars = 20 *μ*m). (d) The data represent the means ± SDs of at least three individual experiments; *∗P*<0.05, *∗∗P*<0.01, *∗∗∗P*<0.001 (in comparison with the PA-treated model group).

Since inflammation is a pivotal factor that exacerbates the evolvement of simple steatosis to nonalcoholic steatohepatitis (NASH) [27], we examined the inflammatory signaling through NF-*κ*B by immunoblotting and quantified the mRNA levels of proinflammatory cytokines to assess the effects of G-Rg1 on the inflammatory status in the liver. IĸB*α* phosphorylation was predominant in cells treated only with PA, in which the p-IĸB*α*/total IĸB*α* ratio was 5.5 times greater than that of the control cells, while treatment with 40 or 80 *μ*g/mL G-Rg1 significantly inhibited IĸB*α* phosphorylation, thus blocking its degradation. Compared with the control group, the phosphorylation of NF-*κ*B (p65) in the model group showed a 1.1-fold increase. Upon treatment with 40 and 80 *μ*g/mL G-Rg1, p65 phosphorylation decreased significantly by 47.6% and 23.8%, respectively (Figures 6(a) and 6(b)). Further, the protein expression levels of pro-IL-1*β*, cleaved IL-1*β*, IL-6, and TNF-*α* were 2.4, 1.7, 2.0, and 2.6 times higher than those in cells from the control group after PA treatment. However, the two concentrations of G-Rg1 reversed the PA-induced inflammation and reduced the expressions of pro-IL-1*β*, cleaved IL-1*β*, IL-6, and TNF-*α* by 50.0%, 35.2%, 47.1%, and 50.0%, respectively (Figures 6(a) and 6(b)). Next, we detected the mRNA levels of proinflammatory cytokines, including IL-1*β*, IL-6, and TNF-*α*, which increased by 3.3, 3.2, and 2.9 times, respectively, in the model group, compared to the case for the control group. Upon treatment with 40 *μ*g/mL G-Rg1, the mRNA levels of IL-1*β*, IL-6, and TNF-*α* decreased significantly by 57.3%, 45.8%, and 36.3%, respectively (40 *μ*g/mL G-Rg1), and by 45.9%, 32.7%, and 54.0%, respectively (80 *μ*g/mLG-Rg1) (Figure 6(c)). When NF-*κ*B is activated, the p65 subunit of the NF-*κ*B complex gets translocated from the cytoplasm to the nucleus. From our immunofluorescence results, it was evident that the GFP-labeled NF-*κ*B p65 subunit was mainly located in the cytoplasm under normal physiological conditions and was translocated to the nucleus when the HepG2 cells were stimulated by 0.25 mM PA. However, low and high doses of G-Rg1 inhibited the nuclear translocation of the NF-*κ*B p65 subunit to varying degrees (Figure 6(d)). These results suggest that G-Rg1 can ameliorate PA-induced liver inflammation.

## 4. Discussion

Nonalcoholic fatty liver disease (NAFLD) has attained extensive attention on account of its high morbidity, heavy economic burdens, and tremendous mental pressure. However, a lack of therapeutic strategies renders it difficult to treat. Thus, it is imperative to find new remedies to treat NAFLD [28]. Studies have demonstrated that lipid deposition, oxidative stress, lipid peroxidation, and inflammatory responses are involved in the development of NAFLD. Notably, these mechanisms crosstalk with each other, and inflammatory response plays an important crosslinking role in the evolution of NAFLD [20]. It has been reported that G-Rg1 can improve lipid peroxidation, promote fatty acid *β*-oxidation, and inhibit inflammasome activation in mice with high-fat diet-induced NAFLD [25]. However, there are no studies targeting the impact and mechanism of G-Rg1 on lipid metabolism and inflammatory responses in NAFLD. Thereafter, the aim of our present investigation was to explore the potential capacity and target spot of G-Rg1 to ameliorate lipid deposition and inflammation for NAFLD treatment.

Palmitic acid (PA) is one of the most abundant saturated fatty acids in our daily diets; it is known to cause lipotoxicity by multifaceted mechanisms [29]. In this study, a NAFLD cell model was successfully established with 0.25 mM PA, and then 40 and 80 *μ*g/mL G-Rg1 were used to investigate the effects of G-Rg1 in HepG2 cells. As expected, we found that both concentrations could ameliorate PA-induced lipid accumulation, and the effect of 80 *μ*g/mL G-Rg1 was more significant than that of 40 *μ*g/mL (Figure 2). Likewise, the cell supernatant levels of aminotransferases, which have long been considered indicators of hepatic injury, were drastically increased following PA administration, while G-Rg1 exerted a protective effect against PA-induced liver injury (Table 2). It is widely accepted that Kupffer cells are an important source of proinflammatory cytokines, such as IL-1*β*, IL-6, and TNF-*α* in the liver. Earlier studies have primarily focused on the inflammatory pathways of Kupffer cells and ignored hepatocytes. Interestingly, results obtained using hepatocytes have displayed that the levels of proinflammatory mediators were elevated dramatically in fatty livers [30]. Furthermore, Panahi G et al. have reported that high glucose concentrations increased the expression of cytokines in HepG2 cells [31]. Our* in vitro* results also demonstrated that PA prominently increased IL-1*β*, IL-6, and TNF-*α* release in the HepG2 cell supernatants, which were then reduced by G-Rg1 (Figure 3).

As an energy sensor, AMPK systematically adjusts the metabolic energy balance; thus, it is considered as a latent target for treating metabolic diseases [32]. It is indicated that AMPK activity is reduced in the adipose tissues of obese rodents and humans, and nutritional interventions reawaken this activity, preventing the progression of obesity [12]. Increasing evidence has shown that G-Rg1 activates AMPK* in vitro* and* in vivo*, such as the case in HepG2 cells and diabetic model mice [20, 33, 34]. Consistent with a previous study, we found that G-Rg1 increased the phosphorylation of AMPK in HepG2 cells. AMPK is the main kinase regulator of its downstream genes ACC*α*, SREBP-1c, and FAS, which play a major role in adipocyte differentiation and lipid management [35]. In our study, it was shown that G-Rg1 suppressed the activity of ACC*α*, SREBP-1c, and FAS through the phosphorylation of AMPK (Figure 4). Additionally, blocking AMPK by using an AMPK inhibitor prominently abolished the G-Rg1-mediated protection against PA-induced lipid deposition in HepG2 cells (Figure 5). Noticeably, this effect was not entirely dose-dependent, we supposed that this obvious discrepancy could be explained by the differences between the kinetics of phosphorylation and its downstream reaction associated with the two concentrations. Specifically, we confirm that phosphorylation occurs more expeditiously and transiently in case of high G-Rg1 concentrations than low G-Rg1 concentrations, which emphasizes the importance of choosing an optimal time-period for detecting subtle cellular changes.

Furthermore, we investigated the role of G-Rg1 in the regulation of NF-*κ*B activity. Previous studies have indicated that NF-*κ*B regulates the release of proinflammatory cytokines, and the blockade of NF-*κ*B activation can decrease the expression of proinflammatory factors [36, 37]. Gao et al. also demonstrated that G-Rg1 inhibited inflammation effectively through the inhibition of glucocorticoid receptor-dependent NF-*κ*B activity [38]. Similarly, our present study demonstrated that G-Rg1 reduced IĸB*α* degradation, suppressed NF-*κ*B p65 phosphorylation, and prevented the translocation of p65 into the nucleus, where its gene expression is regulated. These variations were in accordance with the observed changes of cytokine expressions (Figure 6). Thus, we speculated that the attenuation of the severity of PA-induced liver inflammation by G-Rg1 is mediated by the downregulation of the NF-*κ*B activity. However, the activity of kinases located upstream of NF-*κ*B, such as IKK, is not disclosed. Intriguingly, there are emerging results indicating that AMPK might inhibit inflammatory responses via suppressing the activity of NF-*κ*B, but there is currently no evidence indicating that any of the NF-*κ*B subunits or the kinases located upstream of the NF-*κ*B are the direct phosphorylation targets of AMPK [39–41]. To dig deeper into the links between AMPK and NF-*κ*B and to clarify whether G-Rg1 ameliorates inflammation through the direct inactivation of NF-*κ*B or the indirect activation of AMPK, small interfering RNAs should be utilized, and the upstream genes should be detected simultaneously in the further studies to provide ample evidence clarifying these aspects.

Collectively, we found that G-Rg1 could mitigate the formation of lipid droplets and ameliorate inflammation in PA-treated HepG2 cells. These beneficial effects might be ascribed to the AMPK/NF-*κ*B pathway. Although the potential off-target effects of G-Rg1 may be considered and more definitive evidence should be obtained using genetic approaches, our findings indicate that G-Rg1 may be a novel potential therapeutic agent for the treatment of NAFLD. Additionally, further investigations are required to thoroughly elucidate the mechanisms and clinical applications of this natural agent both* in vitro* and* in vivo*.

## Figures and Tables

**Table 1:** Sequences of primers used for quantitative real-time PCR.

Gene	Sequence (5'-3')
SREBP-1c (human)	Forward: CGGAACCATCTTGGCAACAGT
	Reverse: CGCTTCTCAATGGCGTTGT
FAS (human)	Forward: AGATTGTGTGATGAAGGACATGG
	Reverse: TGTTGCTGGTGAGTGTGCATT
IL-1*β* (human)	Forward: ATGATGGCTTATTACAGTGGCAA
	Reverse: GTCGGAGATTCGTAGCTGGA
IL-6 (human)	Forward: ACTCACCTCTTCAGAACGAATTG
	Reverse: CCATCTTTGGAAGGTTCAGGTTG
TNF-*α* (human)	Forward: GAGGCCAAGCCCTGGTATG
	Reverse: CGGGCCGATTGATCTCAGC
*β*-actin (human)	Forward: GCCGACAGGATGCAGAAGG
	Reverse: TGGAAGGTGGACAGCGAGG

**Table 2:** *Levels of transaminases in cell supernatants*. HepG2 cells were treated with 0.25 mM PA for 24 h and then cultivated with 40 or 80 *μ*g/mL G-Rg1 for 6 h. The data are presented as means± SDs (n=3). *∗∗*p<0.01 (in comparison to the PA-treated model group).

Parameters(U/L)	Control	PA	PA+40*μ*g/mL G-Rg1	PA+80*μ*g/mL G-Rg1
ALT	31.72±3.40*∗∗*	91.37±7.37	70.2±6.19 *∗∗*	58.5±4.15*∗∗*
AST	7.40±0.97 *∗∗*	11.7±1.36	8.93±0.86*∗∗*	7.43±0.82*∗∗*

## Data Availability

The data used to support the findings of this study are available from the corresponding author upon request.

## Supplementary Materials

Supplementary Materials Figure 7. Schematic representation of the beneficial effects of ginsenoside Rg1 (G-Rg1) on NAFLD cell model. G-Rg1 inhibits lipid accumulation and reduces biochemical indicators and the release of proinflammatory factors in HepG2 cells, which is related to AMPK/NF-kB pathway.
