# Supplementary figures and images for: Ginsenoside Rg1 Ameliorates Palmitic Acid-Induced Hepatic Steatosis and Inflammation in HepG2 Cells via the AMPK/NF-κB Pathway
Source: Int J Endocrinol. 2019 Jul 28;2019:7514802. doi: 10.1155/2019/7514802 (PMC6699274; doi:10.1155/2019/7514802)

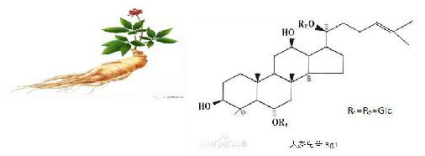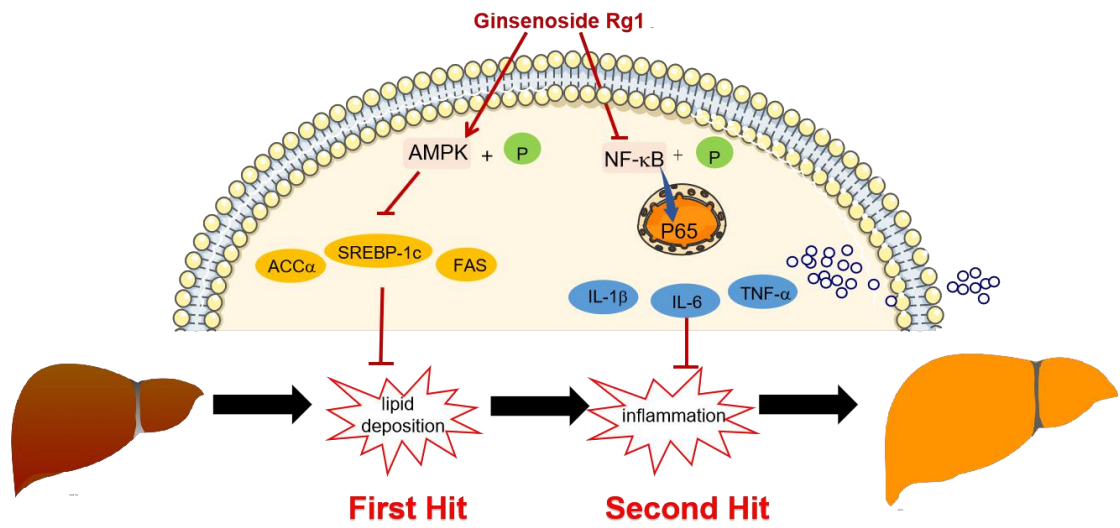

Supplement: Supplementary Materials — Figure 7. Schematic representation of the beneficial effects of ginsenoside Rg1 (G-Rg1) on NAFLD cell model. G-Rg1 inhibits lipid accumulation and reduces biochemical indicators and the release of proinflammatory factors in HepG2 cells, which is related to AMPK/NF-kB pathway. [file 7514802.f1.pdf]
